# Adult height and incidence of atrial fibrillation and heart failure in older men: The British Regional Heart Study

**DOI:** 10.1016/j.ijcha.2021.100835

**Published:** 2021-07-08

**Authors:** S. Goya Wannamethee, Olia Papacosta, Lucy Lennon, Aroon Hingorani, Peter Whincup

**Affiliations:** a Department Primary Care and Population Health, UCL London, United Kingdom; b Institute of Cardiovascular Sciences, UCL, London, United Kingdom; c Population Health Research Institute, St George’s, University of London, United Kingdom

**Keywords:** Height, Atrial fibrillation, Heart failure, Epidemiology, AF, atrial fibrillation, CRP, C-reactive protein, hsTnT, high sensitive troponin T, CHD, coronary heart disease, CVD, cardiovascular disease, ECG, electrocardiogram, FEV_1_, forced expiratory volume in 1 s, HF, heart failure, LVH, left ventricular hypertrophy, MI, myocardial infarction, NT-proBNP, N-terminal pro-brain natriuretic peptide, SBP, systolic blood pressure

## Abstract

**Aims:**

Taller stature has been associated with increased risk of atrial fibrillation (AF). AF and heart failure (HF) often co-occur but the association between height and risk of HF in older adults has not been well studied. We have examined the association between height and incident AF and incident HF in older adults.

**Methods:**

Prospective study of 3346 men aged 60–79 years with no diagnosed HF, myocardial infarction or stroke at baseline (1998–2000) followed up for a mean period of 16 years, in whom there were 294 incident HF cases and 456 incident AF. Men were divided into 5 height groups: <168.2, 168.2–172.5, 172.6–176.9, 177.0–183.0 and >183.0 cms based on the 25th, 50th, 75th and 95th centiles distribution of height.

**Results:**

CVD risk factors tended to decrease with increasing height but a positive association was seen between height and electrocardiographic QRS duration and incident AF. Both short stature (<168.2 cm) and tall stature (>183.0 cm) was associated with significantly increased risk of HF in age-adjusted analysis compared to those in the second height quartile [HR (95 %CI) = 1.62 (1.15, 2.26) and 2.04 (1.23, 3.39) respectively]. In short men the increased risk remained after adjustment for adverse CVD risk factors; in tall men the association was largely associated with AF and QRS duration.

**Conclusion:**

Tall stature is associated with significantly increased risk of AF leading to increased risk of HF. Short stature was associated with increased HF risk which was not explained by known adverse CVD risk factors.

## 1 Introduction

Atrial fibrillation (AF) is the most common heart rhythm disorder in older adults and is strongly associated with heart failure (HF) [1]. Although short stature has been associated with increased risk of coronary heart disease [2], previous observational studies have consistently shown tall stature to be associated with increased risk of AF [3], [4], [5], [6], [7], [8], [9] and a recent large Mendelian Randomization Study suggest that the association is causal [10]. Moreover, recent studies have reported associations between tall stature and abnormal electrocardiographic repolarisation markers such as the QRS duration [11], [12], [13] which have been associated with incident AF and HF [14], [15], [16], [17]. Relatively few studies in western populations have investigated the association between height and development of HF and in the few that have the findings have been inconsistent. In middle-aged populations, two studies showed an inverse association between height and incident HF risk [7], [18], while one study reported no significant association between height and HF although the highest risk was observed in tall adults [19]. The lower risk of HF in tall adults reported in middle-aged populations may reflect the reduced risk of coronary heart disease associated with greater height [2]. Incidence of AF increases steeply with age and is a major contributor to HF in older adults. The inverse association between height and HF risk reported in younger populations may not apply in older people in whom HF is often not preceded by a myocardial infarction (MI). Thus we hypothesised that tall stature would be associated with increased risk of HF in older adults. We have therefore examined prospectively the association between adult height and incident AF and incident HF in a population based cohort of older men.

## 2 Subjects and methods

The British Regional Heart Study is a prospective study of cardiovascular disease involving 7735 men aged 40–59 years drawn from general practice in each of 24 British towns, who were screened between 1978 and 1980 [20]. The population studied was socio-economically representative of British men but consisted almost entirely of white Europeans (>99%). In 1998–2000, all surviving men, then aged 60–79 years (mean age 68.7 years), were invited for a 20th year follow-up examination and 4252 men (77% of survivors) attended for examination. The men completed a questionnaire which included questions on their medical history and lifestyle behaviour. They were requested to fast for a minimum of 6 h, during which time they were instructed to drink only water and to attend for measurement at a pre-specified time between 0800 and 1800 h. All men were asked to provide a blood sample, collected using the Sarstedt Monovette system. The protocol complied with the Declaration of Helsinki of 1975 (as revised in 1983) and all relevant local research ethics committees provided ethical approval. In 2010–2012 surviving study members aged 71–92 years (n = 3137) were invited to attend a 30-year re-examination, of whom 1722 attended a physical examination (55% response rate).

### 2.1 Cardiovascular risk factor measurements at 1998–2000

Anthropometric measurements including body weight, height and waist circumference were carried. Details of measurement and classification methods for smoking status, physical activity, social class, alcohol intake, blood pressure and blood lipids in this cohort have been described [21], [22]. Heavy drinking was defined as drinking more than five units (1 UK unit = 10 g) of alcohol daily on most days. The men were also asked to report their pattern of physical activity such as walking, cycling and other sporting activities. Physical activity scores were assigned on the basis of frequency and type of activity and the men were divided into 6 groups: none, occasional, light, moderate, moderately-vigorous and vigorous. Subjects who reported none or occasional activity were classified as ‘inactive’. Social class was derived from the longest held occupation recorded at the time of baseline questionnaire (1978–80) using the Registrar General’s classification of occupations, with categories grouped as non-manual (I, II and III non-manual) and manual (III manual, IV and V). Predicted glomerular filtration rate (eGFR) (measure of renal function) was estimated from serum creatinine using the equation eGFR = 186 × (creatinine)-1.154 × (age)-0.203 [23]. Renal dysfunction was defined as eGFR < 60. N-terminal pro-brain natriuretic peptide (NT-proBNP) and high sensitive troponin T (hsTnT) were determined using the Elecsys 2010 electrochemiluminescence method (Roche Diagnostics, Burgess Hill, UK) [24].

### 2.2 ECG measurements

At both re-examinations in 1998–2000 and 2010–2012 12 lead electrocardiograms were recorded using a Siemens Sicard 460 instrument and were analyzed at the University of Glasgow ECG core laboratory using Minnesota Coding definitions [25]. Prolonged QRS duration was defined as values > 100 ms and abnormal as >=120 ms [26]. Electrocardiographic left ventricular hypertrophy (LVH) was defined according to relevant Minnesota codes (codes 3.1 or 3.3). AF was defined according to Minnesota codes 8.3.1 and 8.3.3.

### 2.3 Follow-up

All men have been followed up from initial examination (1978–1980) for cardiovascular morbidity [20] and follow-up has been achieved for 99% of the cohort. In the present analyses, all-cause mortality and HF events are based on follow-up from re-examination in 1998–2000 at mean age 60–79 years to June 2016. Survival times ended at the first HF event or when they were censored for death due to any cause, or the end of the follow-up period (June 2016), whichever occurred first. Information on death was provided by the UK National Health Service registers. Fatal CHD events were defined as death with CHD (ICD 9th revision, codes 410–414) as the underlying code. A non-fatal MI was diagnosed according to World Health Organisation criteria. Evidence of non-fatal MI and HF was obtained by ad hoc reports from general practitioners supplemented by biennial reviews of the patients' practice records (including hospital and clinic correspondence) through to the end of the study period. Incident CHD included fatal CHD and non-fatal MI. Incident non-fatal HF was based on a doctor-confirmed diagnosis of HF from primary care medical records (including hospital and clinical correspondence) [24]. All cases were verified by a review of available clinical information from primary and secondary care records (symptoms, signs, investigations, and treatment response) to ensure they are consistent with current recommendations on HF diagnosis [27]. Incident fatal HF cases were those in which the diagnosis of HF was mentioned as the underlying cause of death at death certificates (ICD 9th revision code 428). Incident HF included both incident non-fatal HF and incident fatal HF.

#### 2.3.1 Incident Atrial fibrillation

Incident AF was obtained from ad hoc reports from general practitioners supplemented by reporting of medication for AF by the men on follow-up questionnaires or evidence of AF on ECG at the 30-year examination. The men were asked about medication and the reason for taking the medication. Only men who reported medication for treatment of AF specifically were included**.** Patient reporting of medication for AF was confirmed in 77% of cases.

### 2.4 Statistical analysis

The men were initially divided into 4 groups based on quartiles of the height distribution. Because of the interest in very tall adults we separated out the top 5% of men in the height distribution and 5 height groups were used. Cox's proportional hazards model was used to assess the multivariate-adjusted hazards ratio (relative risk) for HF and incident AF in a comparison of 5 height groups: <168.2, 168.2–172.5, 172.6–176.9, 177.0–183.0 and > 183.0 cms using the 2nd quartile as the reference group. Restricted cubic splines were used to visually depict the association between height and incident HF**.** In multivariate analyses, smoking, social class, physical activity, alcohol intake, diabetes, use of antihypertensive treatment, renal dysfunction were fitted as categorical variables; CRP, systolic blood pressure, waist circumference and QRS duration were fitted as continuous variables. Adjustment for incident AF was fitted as a time dependent covariate. In the analysis assessing height and incident AF, all men with prevalent AF (N = 78) were excluded.

## 3 Results

From the 4252 men examined in 1998–2000 we excluded men with doctor diagnosed MI, stroke or HF. After these exclusions 3346 men were available for analysis. During the average follow-up period of 16 years there were 294 incident HF cases in the 3346 men with no previous history of HF, MI or stroke.

### 3.1 Baseline characteristics

Table 1 shows baseline characteristics in the study population by the 5 height groups. Short men tended to have more adverse risk factors than taller men. They were older and had the highest rates of smoking, physical inactivity and use of antihypertensive drugs and were more likely to be manual workers. They had the highest levels of systolic blood pressure, inflammation (CRP), vWF, heart rate, NT-proBNP and hsTnT, evidence of ischaemia and the lowest level of lung function. However, positive associations were seen between height, AF and QRS duration, with the tallest men having the highest prevalence of AF and longer mean QRS duration. The positive associations between height and AF and height and QRS duration remained significant even after adjustment for age and BMI and were independent of each other. In mutually adjusted analyses also including age and BMI, the odds of having AF for the tallest men compared to the reference group was 4.52 (1.97,10.37) and the odds of having an abnormal QRS duration was 2.01 (1.22,3.29).

**Table 1:** Baseline characteristics by height in 3346 men without prevalent HF, MI or stroke.

			Height (cm)			
	<168.2	168.2–172.5	172.6–176.9	177–183.0	>183.0	*p-trend*
	N = 841	N = 829	N = 849	N = 650	N = 177	
Age	69.5 (5.7)	68.9 (5.5)	68.1 (5.3)	67.2 (4.9)	66.3 (4.9)	*<0.0001*
WC	94.2 (10.2)	96.3 (9.4)	97.44 (10.1)	98.85 (9.9)	101.6 (11.4)	*<0.0001*
BMI	26.80 (3.79)	26.77 (3.39)	26.80 (3.68)	26.74 (3.43)	26.77 (3.53)	*0.78*
% obese	16.6	15.2	15.3	14.9	15.3	*0.20*
% smokers	15.6	12.9	10.8	13.6	6.9	*0.009*
% manual workers	65.5	55.8	50.2	42.2	37.1	*<0.0001*
% heavy drinking	3.8	3.8	3.7	4.6	4.0	*0.64*
% inactive	36.7	33.8	30.8	27.7	31.0	*0.0002*
% BP loweringdrugs	29.5	25.4	27.6	23.8	25.7	*0.06*
% diabetes	11.8	11.2	11.8	12.5	13.1	*0.48*
% CKD	17.0	13.4	13.9	10.5	15.4	*0.01*
**Biological markers**						
SBP (mmHg)	152.1 (24.5)	149.8 (24.5)	149.9 (23.3)	148.9 (22.6)	146.0 (23.1)	*0.001*
Cholesterol (mmol/l)	6.10 (1.10)	6.08 (1.06)	6.09 (1.05)	6.02 (1.05)	5.82 (0.92)	*0.01*
HDL-C (mmol/l)	1.35 (0.35)	1.32 (0.33)	1.34 (0.35)	1.32 (0.33)	1.32 (0.34)	*0.12*
CRP (mg/L)*	1.90	1.68	1.58	1.51	1.36	*<0.0001*
vWF ((IU/dl)	143.2 (46.5)	139.0 (44.4)	135.3 (46.0)	131.2 (42.0)	132.4 (47.1)	*<0.0001*
FEV1 (L)	2.31 (0.61)	2.56 (0.65)	2.70 (0.65)	2.88 (0.72)	3.11 (0.72)	*<0.0001*
Heart Rate (b/min)	66.9 (12.0)	66.7 (13.3)	64.9 (12.2)	65.3 (12.9)	63.6 (13.2)	*<0.0001*
NT-proBNP (pg/ml)*	95.6	81.58	82.3	83.1	83.1	*0.04*
hsTnT (pg/ml)*	12.06	11.58	11.47	11.02	11.02	*0.0002*
**ECG parameters**						
% AF	2.8	2.0	3.1	2.6	5.7	*0.18*
% LVH	8.4	8.7	6.7	6.2	5.1	*0.02*
% evidence ischaemia	25.2	23.0	18.3	19.6	17.7	*0.001*
QRS duration	9.69 (1.67)	9.89 (1.83)	10.05 (1.81)	10.05 (1.64)	10.32 (1.85)	*<0.0001*
%>100 ms	22.2	27.8	34.1	35.2	54.3	*<0.0001*
%≥120 ms	6.8	9.3	10.6	9.1	13.7	*<0.0001*

* Geometric mean

### 3.2 Height and incident AF

There were 456 incident AF cases in the 3268 men with no prevalent AF. Table 2 shows the association between height and incident AF. Risk of developing AF increased with increasing height with the tallest men having nearly a two fold increase in risk of developing AF compared to the reference group. This was seen even after adjustment for a wide range of possible confounders including age, smoking, physical inactivity, heavy drinking, social class, waist circumference, blood pressure, cholesterol, FEV1, renal function, diabetes, use of antihypertensive drugs, LVH and CRP and QRS-duration.

**Table 2:** Height and adjusted hazards ratio (95 %CI) incident AF in 3268 men with no prevalent AF, HF, stroke or MI.

				HR (95 %CI)		
			Height (cms)			
	<168.2	168.2–172.5	172.6–176.9	177.0–183.0	>183.0	Linear trend
No of men	822	817	827	655	167	
Incident AF % (n)	8.3 (68)	13.6 (1 1 1)	14.2 (1 1 7)	19.4 (1 2 3)	22.2 (37)	
Age-adjusted	0.63 (0.47,0.85)	1.00	1.03 (0.79,1.33)	1.45 (1.12,1.87)	1.75 (1.20,2.54)	<0.0001
Model 1	0.65 (0.48,0.89)	1.00	1.05 (0.80,1.36)	1.37 (1.05,1.78)	1.71 (1.13,2.51)	<0.0001
Model 2	0.67 (0.49,0.91)	1.00	1.05 (0.80,1.37)	1.35 (1.05,1.80)	1.65 (1.13,2.45)	<0.0001

Model 1 Adjusted for age, smoking, physical inactivity, heavy drinking, social class, blood pressure, waist circumference, cholesterol, FEV1, renal function, diabetes, use of antihypertensive drugs, LVH and CRP.

Model 2 Model 1 + QRS duration

We carried out a sensitivity analysis restricted to those with ECG evidence of AF based on a subgroup of men (n = 1348) who attended both the 1998–2000 and the 2010–12 re-examination. The results confirmed the highest risk of AF seen in the tallest men.

### 3.3 Height and incident HF

**Fig. 1:**
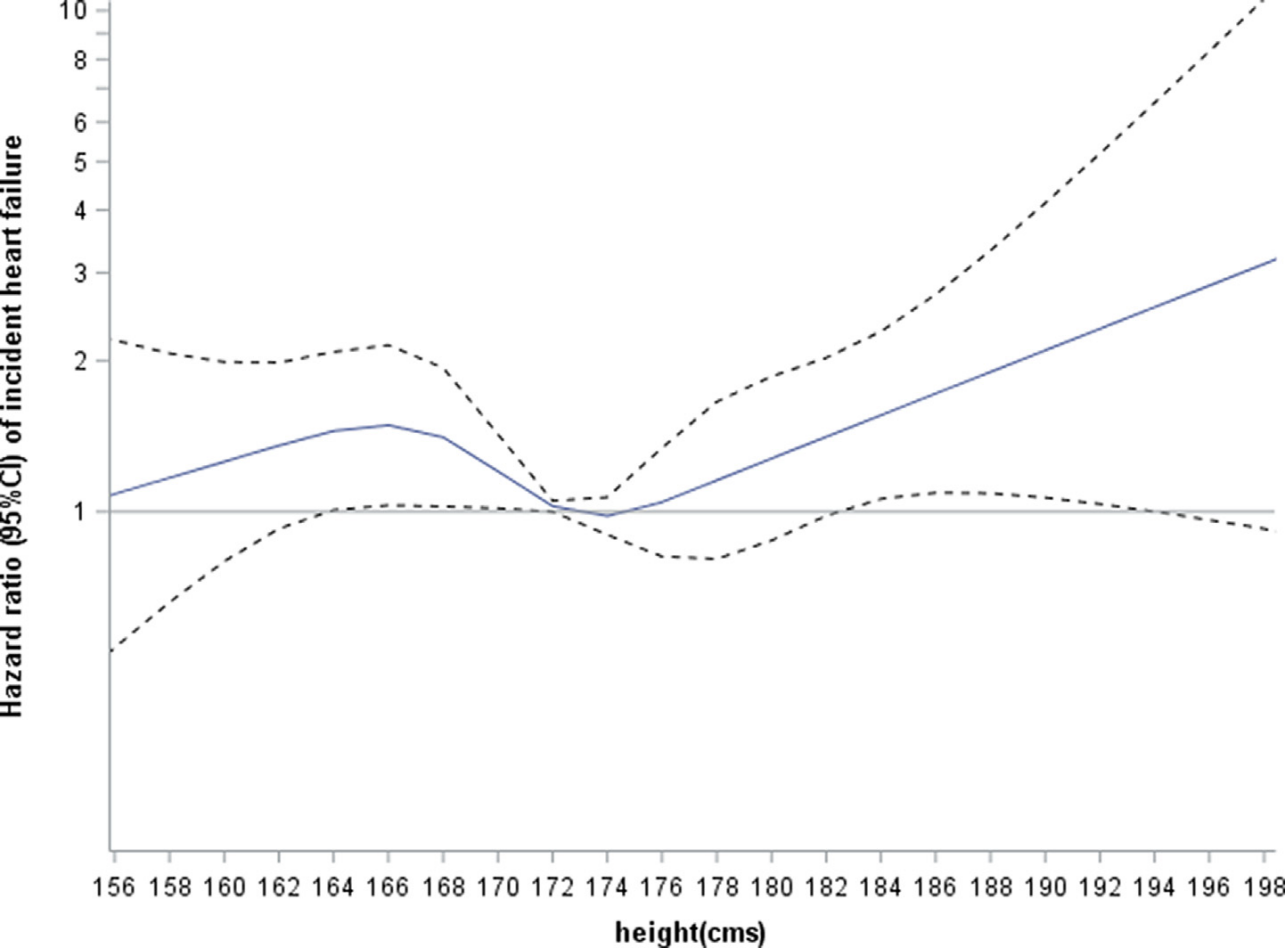
Association of height (cms) with risk of incident heart failure: height modelled as restricted cubic splines with knots at the 5th (162cms) 25th, 50th, 75th and 95th (183 cms) percentiles adjusted for age, smoking, physical activity, social class, waist circumference, diabetes, antihypertensive treatment, eGFR, LVH, heavy drinking, systolic blood pressure and CRP.

In age-adjusted analysis, tall stature in particular, as well as short stature was associated with significantly increased risk of HF with the lowest risk in men in the second quartile (Table 3). The increased risk seen in short men remained after adjustment for lifestyle characteristics, established CHD risk factors and CRP. Since tall men had the most favourable CHD risk factors, adjustment increased the risk further (Table 3; model 1). Fig. 1 shows the continuous association between height and risk of HF after these adjustments. Further adjustment for prevalent AF and QRS duration attenuated the increased risk seen in tall men (Table 3; models 2 and 3). Of the men who developed HF during follow-up 58 men (20%) had developed AF prior to the HF event. Comparisons between the height groups showed that 11.4% (n = 10) of HF cases was preceded by incident AF in short men compared with 33.3% of cases (n = 7) in the tall men. Thus the increased risk of HF in the tall men was attenuated further after taking into account men who developed AF (Table 3; model 4). Short stature remained significantly associated with increased risk of HF after adjustment and was strengthened after adjustment for incident AF.

**Table 3:** Incidence rates/1000 person years and adjusted relative hazard ratios (HR) and 95% CI for incident HF by height in 3346 men with no prevalent HF, stroke or MI.

			Height (cms)		
	<168.2 (N = 841)	168.2–172.5 (N = 829)	172.6–176.9 (N = 849)	177.0–183.0 (N = 650)	>183.0 (N = 177)
Rate/1000 (n)	8.8 (88)	5.1 (55)	6.6 (74)	6.1 (55)	8.8 (21)
Age adjusted	1.62 (1.15,2.26)	1.00	1.34 (0.94,1.89)	1.32 (0.91,1.92)	2.04 (1.23,3.39)
Model 1	1.65 (1.17,2.33)	1.00	1.35 (0.94,1.72)	1.25 (0.85,1.84)	1.87 (1.11,3.15)
Model 2	1.62 (1.14,2.30)	1.00	1.31 (0.92,1.88)	1.22 (0.83,1.80)	1.76 (1.04,2.96)
Model 3	1.69 (1.19,2.40)	1.00	1.30 (0.91,1.86)	1.19 (0.81,1.75)	1.63 (0.97,2.76)
Model 4	1.82 (1.28,2.59)	1.00	1.33 (0.92,1.90)	1.14 (0.78,1.69)	1.50 (0.89,2.55)

Model 1 adjusted for age, smoking, physical inactivity, heavy drinking, social class, waist circumference, blood pressure, cholesterol, FEV1, renal function, diabetes, use of antihypertensive drugs , LVH, CRP

Model 2 = Model 1 + prevalent AF

Model 3 = Model 2 + QRS duration

Model 4 = Model 2 + incident AF

### 3.4 Height and incident CHD

There was a curvilinear relationship between height and risk of incident CHD (N = 424 cases) with those in the third quartile (172.6–176.9 cms) having the lowest risk; taller stature was not associated with lower CHD risk. The age-adjusted HR (95 %CI) for the 5 height groups were 1.23 (0.96,1.58), 1.00, 0.75 (0.57,0.99), 1.07 (0.79,1.46) and 0.99 (0.62,1.60) respectively. Adjustment for incident MI in addition to variables in model 1 (Table 3) made minor differences to the elevated risk seen in men with short stature [HR = 1.64 95% CI (1.17, 2.29)]

## 4 Discussion

In this study of older men, we have confirmed previous observational studies on the association between tall stature and increased risk of incident AF [3], [4], [5], [6], [7], [8], [9]. AF is strongly associated with HF and our findings extend previous studies by examining whether the association with height may influence HF risk in older tall adults. We have shown that both short stature and tall stature are associated with increased HF risk but the pathways underlying these associations may be different. The increased risk of HF in short adults was not explained by adverse CVD risk factors associated with short stature. By contrast, the increased risk of HF in tall men was largely explained by their increased risk of AF and prolonged QRS duration, suggesting mechanisms linked to left ventricular damage and the cardiac conduction system [11], [12], [13].

### 4.1 Height and incident AF

In line with other observational studies we have shown tall stature to be associated with increased risk of AF [3], [4], [5], [6], [7], [8], [9] and this was independent of known confounders including BMI and LVH. Indeed height has already been included in clinical prediction score for AF [3]. Adult height results from a combination of genetic and environmental factors. It is relatively unclear how tall stature is related to incident AF. It is suggested that the association may be linked to the size of the left atrium of the heart which is known to be strongly associated with body size and height and a major risk factor for the development of AF [5], [28], [29]. We did not have echocardiographic measurements. However, two previous reports have shown the association between height and AF to be independent of left atrial size [4], [5], [29] suggesting other pathophysiologic mechanisms. The association between height and AF may be related to premature atrial contractions, a strong predictor of AF and associated complications [30]. Both small-scale and large scale genetic studies have also shown associations between height associated genetic variants and AF suggesting that the association is causal [10], [11], [13] and may involve mechanisms associated with growth pathways and AF.

### 4.2 Height and electrocardiographic QRS duration

Height was significantly associated with the QRS duration, a marker of ventricular depolarisation, as measured on the 12-lead electrocardiogram. This finding is consistent with previous reports showing QRS duration to be associated with greater body height [11], [12]. There is increasing evidence that a prolonged QRS duration on the ECG is associated with increased risk of HF [14], [15], [16], [17]. A QRS duration < 100 ms has been considered normal. Over 50% of the tall men who had no history of CVD or heart failure had QRS duration > 100 ms compared to 22–35% in the other height groups and they had the highest prevalence of men with a QRS duration >=120 ms which is traditionally considered abnormal [26]. QRS duration ≥ 120 ms may be a marker of increased left ventricular (LV) mass but can also suggest functional abnormalities such as LV dysfunction [26]. QRS prolongation may also be a manifestation of ischemic injury to the myocardium, a common precursor of new HF. However the tall men did not have raised hsTnT or NT-proBNP (markers of cardiac injury). The strong association between tall stature and prolonged QRS duration may reflect the known association between height and LV mass [28].

### 4.3 Height and incident HF

Although tall stature has been linked with incident AF, which is strongly associated with HF, relatively few studies have examined the association between height and incident HF. Despite the positive relationship between height and AF, previous studies have reported an inverse association or no association between height and incident HF. In contrast to previous studies we have shown that both short men and in particular tall men were at increased risk of developing HF. In tall men, the increased risk was largely due their increased risk of developing AF and their increased risk of having prolonged QRS duration. The difference in findings on height and incident HF may be due to the difference in the population age as the two studies reporting an inverse association were conducted in middle-age. Height has been shown to be inversely related to the development of CHD which in turn is associated with reduced risk of developing HF. This was observed in these men in an earlier report when the men were middle aged. Height measured when aged 40–59 yrs was inversely associated with 12 year incident CHD with risk decreasing progressively with increasing height [31]. However in older age (60–79 years) tall stature in these men was not associated with reduced risk of subsequent CHD. Older patients with HF differ from younger patients in that a higher proportion of older HF patients have HF with preserved ejection fraction [32]. These patients are less likely to have CHD and more likely to have hypertension and atrial fibrillation which becomes more prevalent in older adults [32]. Thus, despite the increased risk of AF associated with taller stature seen in middle-aged populations, the development of HF may not manifest until later life. A high proportion of men without prior MI in this study who developed HF did not have an MI before developing HF (85%), which could explain the difference in findings between this and the younger US cohorts previously studied. In contrast, CHD is the predominant risk factor for HF with reduced ejection fraction, which is more common in younger adults [32]. This may explain the overall lower risk of HF associated with tall stature seen in these two previous studies conducted in younger populations. Moreover, we have observed that risk is significantly increased only in men over 183cms (6ft), which represented only a small proportion of men aged 60–79 in this cohort of men born between 1919 and 1938. This may also explain the absence of increased risk of HF in the recent Korean study where the height in the top decile of the population was only 170cms [33]. Average body height has increased over the decades [2]; if this trend continues, the prevalence of tall older adults is likely to increase which may contribute to an increasing burden of AF and HF.

By contrast short stature was associated with increased HF despite lower risk of AF and this increased risk was not explained by known adverse CVD risk factors. It is well documented that short stature is associated with increased risk of CHD a major determinant of HF [2]. However the association was not explained by incident MI. The increased risk of HF in short people has been observed in previous studies [33]. The physiological effect of short stature on central haemodynamics and arterial tree may be an explanation for the independent association between short stature and HF [17], [34]. Shorter adults have faster heart rates and increased central aortic pressure leading to increased cardiac overloading and diastolic dysfunction [34]. Short stature has also shown to have physiological effects on the arterial tree, which increase left ventricular pulsatile work and left ventricular mass and jeopardize myocardial perfusion leading to a reduced venous flow to the heart [17], [34]. Alternatively, although adverse risk factors and adult social class did not explain the increased risk in men with short stature it is possible that the increased risk may be due to factors associated with early life circumstances which determines height. The increased risk may be explained by larger differences in social and economic status in the time of the 20–40 s such as poorer hygiene and nutritional conditions in shorter people compared to taller people rather than short status per se.

## 5 Strengths and limitations

The study population is socially representative of the UK population, and follow-up rates in the British Regional Heart Study are very high [20] However it was based on an older predominantly white male population of European extraction, so that the results cannot be generalized directly to women, younger populations or other ethnic groups. The current findings are based on doctor diagnosed HF, which is likely to underestimate the true incidence of HF in this study population. However, the determinants of HF in this study population (including obesity, NT-proBNP and lung function) [21], [22], [24] generally accord with prior data and suggest that the HF outcome used was valid. However, echocardiographic measurements were not routinely carried out and we were not able to differentiate systolic and diastolic HF. Our data was based on doctor diagnosis of AF; tall people tend to be of higher social classes and may be more likely to have their AF diagnosed. Thus the magnitude of effect of tall stature on incident AF may have been overestimated. However, in a subset of men, taller people were also more likely to develop AF on ECG at re-examination. Incident AF was based on doctor diagnosis and evidence on ECG at the two examination periods therefore we may not have detected those with paroxysmal AF. However our findings accord with studies where ECG was carried out annually [4]. Moreover the findings are consistent with prior reports on height and AF in older adults [4], [8] and with previous studies using follow-up hospital registry records [7]. Moreover adjustment for incident AF did attenuate the association between tall stature and incident HF in this study.

## 6 Conclusion

The study confirms the strong positive association between height and increased risk of AF in older age. Tall stature is associated with increased risk HF in older adults which is to a large extent explained by their increased risk of developing AF. Height may serve as a marker for increased AF. Although adult height is not a modifiable factor, height could be taken into account in risk assessment of AF to improve screening for AF in older people, which in turn may help decrease the risk of developing HF in tall older adults.

## Funding Sources

The British Regional Heart Study is a British Heart Foundation (BHF) research group and receives support from BHF Programme grant RG/19/4/34452.

## Declaration of Competing Interest

The authors declare that they have no known competing financial interests or personal relationships that could have appeared to influence the work reported in this paper.
